# Kynurenine as a Predictor of Long-Term Mortality: A 10-Year Follow-Up from the KORONEF Registry

**DOI:** 10.3390/biomedicines13051123

**Published:** 2025-05-06

**Authors:** Adam Kern, Tomasz Stompór, Krystian Bojko, Ewa Sienkiewicz, Sebastian Pawlak, Krystyna Pawlak, Dariusz Pawlak, Grzegorz Poskrobko, Ewa Andrasz, Leszek Gromadziński, Rakesh Jalali, Dariusz Onichimowski, Grażyna Piwko, Artur Zalewski, Jacek Bil

**Affiliations:** 1 Department of Cardiology and Internal Medicine, School of Medicine, Collegium Medicum, University of Warmia and Mazury in Olsztyn, 10-727 Olsztyn, Poland; krystian.bojko@uwm.edu.pl (K.B.); sebastian.pawlak@uwm.edu.pl (S.P.); leszek.gromadzinski@uwm.edu.pl (L.G.); 2 Department of Cardiology, Regional Specialist Hospital in Olsztyn, 10-045 Olsztyn, Poland; stewa@mp.pl (E.S.); gposkrobko@gmail.com (G.P.); ewa8801@wp.pl (E.A.); 3 Department of Nephrology, Hypertension and Internal Medicine, School of Medicine, Collegium Medicum, University of Warmia and Mazury in Olsztyn, 10-727 Olsztyn, Poland; stompin@mp.pl; 4 Department of Monitored Pharmacotherapy, Medical University of Bialystok, 15-089 Bialystok, Poland; krystyna.pawlak@umb.edu.pl; 5 Department of Pharmacodynamics, Medical University of Bialystok, 15-089 Bialystok, Poland; dariusz.pawlak@umb.edu.pl; 6 Department of Emergency Medicine, School of Medicine, Collegium Medicum, University of Warmia and Mazury in Olsztyn, 10-727 Olsztyn, Poland; rakesh.jalali@uwm.edu.pl (R.J.); dariusz.onichimowski@uwm.edu.pl (D.O.); 7 Clinical Emergency Department, Regional Specialist Hospital in Olsztyn, 10-045 Olsztyn, Poland; 8 Clinical Department of Anaesthesiology and Intensive Care, Regional Specialist Hospital in Olsztyn, 10-727 Olsztyn, Poland; 9 Department of Cardiology, University of Warmia and Mazury in Olsztyn, Branch in Ełk, 19-300 Ełk, Poland; grazyna.piwko@uwm.edu.pl; 10 Scanmed Cardiology Center in Ełk, 19-300 Ełk, Poland; artur.zalewski@scanmed.pl; 11 National Medical Institute of the Ministry of Interior and Administration, 02-507 Warsaw, Poland

**Keywords:** kynurenine, tryptophan, cardiovascular disease, mortality, coronary angiography, biomarkers, inflammation, long-term outcomes

## Abstract

**Background**: The kynurenine (KYN) pathway of tryptophan metabolism has been linked to inflammation and cardiovascular risk, but its long-term prognostic value remains unclear. **Methods**: We analyzed 492 patients from the KORONEF registry who underwent coronary and renal angiography and were followed for a median of 10.2 years. Plasma levels of tryptophan (TRP), KYN, and downstream metabolites were measured. The primary endpoint was all-cause mortality. **Results**: The mean age was 64.4 ± 9.9 years, and 37.2% of patients were female. Common comorbidities included hypertension (74.8%), dyslipidemia (46.0%), and diabetes (25.8%). Overall mortality reached 29.5% and increased across KYN tertiles: 17.6% (T1), 28.2% (T2), and 42.9% (T3) (*p* < 0.001). In a multivariable Cox analysis, KYN independently predicted mortality (HR: 1.79; 95% CI: 1.15–2.44; *p* < 0.001), alongside age, diabetes, prior myocardial infarction, chronic kidney disease, and left ventricular ejection fraction. Other kynurenine pathway metabolites were not independently associated with outcomes. **Conclusions**: Elevated kynurenine levels independently predict 10-year all-cause mortality in patients undergoing coronary angiography. KYN may represent a useful prognostic biomarker beyond traditional clinical and angiographic variables.

## 1. Introduction

Cardiovascular diseases (CVDs) remain the leading cause of mortality globally, accounting for an estimated 17.9 million deaths annually. Among these, coronary artery disease (CAD) is the most prevalent form, significantly contributing to the global health burden. Despite advancements in diagnostic and therapeutic strategies, the long-term prognosis of patients with suspected or confirmed CAD varies widely, influenced by a complex interplay of traditional risk factors, comorbidities, and underlying molecular mechanisms [1,2].

While atherosclerosis remains the primary underlying pathology in CAD, an increasing number of patients present with ischemic symptoms or myocardial injury despite having non-obstructed coronary arteries. This clinical diversity highlights the limitations of anatomic assessment alone and underscores the need for novel biomarkers that reflect systemic pathophysiology and predict long-term outcomes beyond the presence or extent of stenosis [3,4,5].

The kynurenine (KYN) pathway of tryptophan (TRP) metabolism has emerged as a promising candidate in this regard. Tryptophan is metabolized primarily through the action of indoleamine 2,3-dioxygenase (IDO) and tryptophan 2,3-dioxygenase (TDO), generating KYN and downstream metabolites such as kynurenic acid (KYNA), 3-hydroxykynurenine (3-HK), 3-hydroxyanthranilic acid (3-HAA), and anthranilic acid (AA). These metabolites exhibit diverse biological effects—ranging from antioxidant to pro-inflammatory—and have been implicated in endothelial dysfunction, oxidative stress, and immune dysregulation [6,7,8,9].

Elevated plasma kynurenine levels and an increased KYN/TRP ratio, a surrogate of IDO activity, have been associated with adverse cardiovascular events and mortality in various populations. However, the long-term prognostic significance of KP metabolites in a real-world population referred for coronary angiography, including both atherosclerotic and non-atherosclerotic cases, remains insufficiently explored [10,11,12].

In this study, using data from the KORONEF registry with 10 years of follow-up, we investigated the association between KP metabolites—TRP, KYN, KYN/TRP ratio, 3-HK, KYNA, 3-HAA, and AA—and all-cause mortality. We aimed to determine whether alterations in the kynurenine pathway can serve as independent predictors of long-term prognosis in a clinically heterogeneous cohort of patients evaluated for suspected cardiovascular disease.

## 2. Materials and Methods

### 2.1. Study Design and Study Population

**Figure 1:**
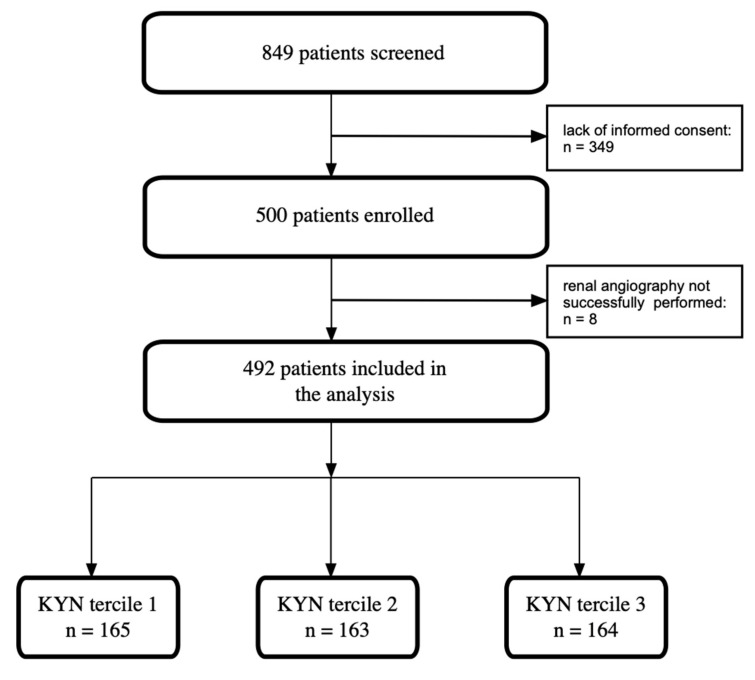
Study flowchart. KYN—kynurenine.

The KORONEF registry was established as a prospective observational study at a single center, as previously documented [13,14]. In brief, the study cohort consisted of 492 consecutive individuals who underwent both coronary and renal angiography (Figure 1).

Most individuals enrolled in the study had been hospitalized for symptoms consistent with coronary artery disease, including stable angina (CCS) and acute presentations: unstable angina (UA), non-ST-elevation myocardial infarction (NSTEMI), and ST-elevation myocardial infarction (STEMI). In addition, the registry encompassed patients referred for coronary angiography with other indications—such as chronic heart failure (HF) or preoperative evaluation prior to cardiovascular procedures, including valve surgery, repair of aortic aneurysm, or implantation of pacemakers or implantable cardioverter defibrillators. The sole exclusion criterion was a lack of informed consent.

Detailed clinical histories were obtained for all participants, with a particular focus on established CAD risk factors. Each patient underwent a physical examination, routine blood tests, and transthoracic echocardiography. All enrolled individuals had coronary and renal artery angiography performed during the same diagnostic session. Based on angiographic findings and clinical assessment, therapeutic strategies were individualized, including optimal medical therapy, percutaneous coronary intervention (PCI), or referral for coronary artery bypass grafting (CABG).

### 2.2. Data Collection

Clinical characteristics were extracted from hospital records and included key comorbidities such as diabetes mellitus, history of smoking, prior myocardial infarction (MI), peripheral arterial disease, dyslipidemia, previous PCI, chronic obstructive pulmonary disease (COPD), arterial hypertension, prior CABG, chronic kidney disease (CKD) defined as an estimated glomerular filtration rate (eGFR) below 60 mL/min/1.73 m^2^, and a history of stroke. In addition, laboratory parameters obtained at the time of admission were assessed.

Quantification of tryptophan and downstream kynurenine pathway metabolites was performed using high-performance liquid chromatography (HPLC). Deproteinized samples were prepared by adding 20 μL 2 M perchloric acid into the 100 μL serum. The acidified samples were vortexed, kept at 4 °C for 20 min, and then centrifuged for 60 min at 12,000 rpm at 4 °C. A total of 2 μL of the supernatant was injected into the HPLC system for analysis. The prepared sample was separated using a Reprospher 100 column (C18, 3.5 μm, 2.1 × 150 mm). Kynurenine (KYN) and tryptophan (TRP) concentrations were measured according to Holmes [15]. The column effluent was monitored with a diode array detector—DAD (KYN-365 nm, TRP-260 nm). The mobile phase was composed of 0.1 M acetic acid and 0.1 M ammonium acetate (pH 4.6) containing 8% acetonitrile, and it was pumped at a flow rate of 0.2 mL/min.

KYNA, AA, and 3-HAA concentrations were measured following the method described by Herve et al. [16], utilizing a Phenomenex PEPTIDE 3.6 μm XB-C18 column (4.6 × 250 mm). Detection was carried out with a programmable fluorescence detector, set to excitation/emission wavelengths of 254/404 nm for all three analytes. The mobile phase, composed of 100 mM zinc acetate and 45 mM acetic acid with 16% acetonitrile, was delivered at a constant flow rate of 0.5 mL/min. A sample volume of 1 μL of the supernatant was injected into the HPLC system for analysis.

3-hydroxykynurenine (3-HK) was measured as described by Heyes [17]. Quantification was performed using a Waters Spherisorb S 3 μm ODS2 column (150 × 2.1 mm), with detection based on electrochemical response. The column effluent was monitored using a programmable electrochemical detector, with the working electrode set to a potential of 0.6 V. The mobile phase consisted of 0.1 M triethylamine, 0.1 M phosphoric acid, 0.3 mM EDTA, and 8.2 mM sodium heptane-1-sulfonate, supplemented with 2% acetonitrile. This phase was delivered at a flow rate of 0.25 mL/min. For each analysis, 2 μL of the sample supernatant was injected into the HPLC system. The detector output was connected to a single instrument—the LC ChemStation.

Also, we documented the pharmacological treatments recommended at hospital discharge, along with echocardiographic measurements. The assessed parameters included left ventricular ejection fraction (LVEF), left ventricular end-diastolic diameter, interventricular septal thickness, right ventricular systolic pressure, and tricuspid annular plane systolic excursion (TAPSE). All echocardiographic evaluations were conducted by experienced cardiologists using a commercially available ultrasound system, in accordance with the standards set by the European Association of Cardiovascular Imaging [18].

Follow-up information was obtained primarily via structured telephone interviews. In cases where direct contact was unsuccessful, data on mortality were supplemented using records from the Central Statistical Office (Główny Urząd Statystyczny, GUS).

### 2.3. Procedure Characteristics

All patients underwent combined coronary and renal artery angiography. Vascular access was obtained via puncture of the right or left femoral artery, and in select cases, the radial artery. Following access, a vascular sheath was introduced to facilitate catheter advancement under fluoroscopic guidance. Coronary arteries were catheterized using standard 6F diagnostic catheters, predominantly with Judkins left (JL4) and right (JR4) curves, and less frequently with Amplatz (AL1, AR1) configurations. Coronary angiography was performed to assess the anatomical distribution, severity, and extent of stenotic lesions, including total occlusions.

Given that femoral access was employed in over 90% of cases, renal angiography could be seamlessly integrated into the procedure. For this purpose, 10–20 mL of contrast agent was administered selectively into each renal artery, allowing for visualization and assessment of potential renal artery stenoses.

### 2.4. Study Endpoints

The primary outcome of the study was all-cause mortality assessed over a 10-year follow-up period. Secondary outcomes included the incidence of MI and stroke, as well as the need for revascularization procedures—either PCI or CABG—within the same decade-long observation window.

### 2.5. Statistical Methods

Initially, in the univariable and then multivariable Cox regression model, we checked the impact of TRP, KYN, KYN/TRP ratio, 3-HK, KYNA, 3-HAA, and AA on long-term outcomes. Only KYN was an independent predictor of all-cause death at ten years.

Descriptive statistics were used to summarize the collected data. Continuous variables were expressed as means with standard deviations (SDs), while categorical variables were reported as counts and percentages. To compare distributions across subgroups, patients were stratified into tertiles based on KYN levels (T1 0.44–1.53 μM; T2 1.54–2.09 μM, and T3 2.10–10.65 μM). For continuous variables, the Kruskal–Wallis test was applied, and for categorical variables, comparisons were made using either Fisher’s exact test or the chi-square test, as appropriate. To control for the risk of type I error in multiple comparisons, a Bonferroni correction was employed to adjust the significance threshold accordingly.

Survival outcomes were assessed using standard time-to-event analysis techniques. Kaplan–Meier curves were generated to estimate survival probabilities, and differences between groups were evaluated using the log-rank test. Both univariable and multivariable Cox proportional hazards regression models were used to assess the association between variables and mortality. Briefly, the multivariable Cox proportional hazards model was constructed by first including all variables that showed a *p*-value < 0.1 in univariable analysis. Then, a backward stepwise elimination procedure was applied using the likelihood ratio test with a removal threshold of *p* > 0.10. To address concerns regarding overfitting, we also performed a sensitivity analysis using a conventional Cox regression model, adjusted only for age, sex, DM, CKD, and prior MI (selected a priori as established predictors). Hazard ratios (HRs) with 95% confidence intervals (CIs) and *p*-values were reported for all models.

All statistical analyses were conducted using R software, version 3.1.2 (R Core Team, 2014), with a significance level set at *p* < 0.05.

## 3. Results

### 3.1. Baseline Characteristics

A total of 492 patients were enrolled in the study between June and December 2011. The cohort included 308 males, accounting for 62.6% of the population. The mean age of participants was 64.4 ± 9.9 years, ranging from 30 to 89 years. The average body mass index (BMI) was 27.9 ± 4.3 kg/m^2^. Baseline characteristics stratified by KYN concentration tertiles are presented in Table 1. Patients in the highest KYN tertile (T3) were significantly older (mean age: 66.0 ± 10.4 years) compared to those in the lower tertiles (*p* < 0.001). A higher prevalence of CKD was observed in T3 (18.4%) relative to T1 (3.6%) and T2 (6.8%) (*p* < 0.001). Prior MI was more frequently reported in T3 (38.7%) compared to T1 (22.4%), with a significant overall trend (*p* = 0.005). Although not statistically significant, trends toward a higher frequency of previous PCI and CABG were noted in the highest tertile. Echocardiographic parameters showed a significant difference in TAPSE, with the lowest mean value observed in T3 (14.5 ± 2.4 mm, *p* = 0.029). Other baseline variables, including BMI, hypertension, diabetes, and LVEF, were not significantly different across tertiles.

The mean TRP concentration was 36.91 ± 11.64 µM, while the average KYN level was 1.93 ± 0.84 µM. The calculated KYN/TRP ratio, an indirect measure of IDO activity, was 0.06 ± 0.04. Among the downstream metabolites of the kynurenine pathway, the mean concentration of 3-HK was 33.44 ± 19.44 nM, while KYNA averaged 26.65 ± 12.37 nM. The mean level of 3-HAA was 22.96 ± 15.13 nM, and AA was present at an average concentration of 31.74 ± 22.42 nM.

Table 2 presents routine biochemical parameters stratified by KYN concentration tertiles. Hemoglobin (Hgb) and glucose values were similar across all groups (*p* = 0.854 and *p* = 0.105, respectively). However, several renal and metabolic markers differed significantly between tertiles. Subjects in the highest KYN tertile (T3) had significantly higher serum creatinine (1.19 ± 0.91 mg/dL) and lower eGFR (70.76 ± 23.41 mL/min/1.73 m^2^) compared to those in the lowest tertile (T1: 0.85 ± 0.20 mg/dL and 90.37 ± 23.26 mL/min/1.73 m^2^, respectively; both *p* < 0.001), indicating a relationship between elevated KYN and impaired renal function. Electrolyte analysis revealed a progressive increase in serum potassium (K^+^) from T1 to T3 (*p* = 0.001), alongside a small but significant reduction in sodium (Na^+^) levels (*p* = 0.027) in the highest tertile.

Total cholesterol (TC) and low-density lipoprotein (LDL-C) were significantly lower in T3 compared to T1 (*p* < 0.001 and *p* = 0.020, respectively), and high-density lipoprotein (HDL-C) levels also decreased across tertiles (*p* = 0.036). Triglyceride (TG) levels showed no significant variation (*p* = 0.234). Inflammatory status, as measured by high-sensitivity C-reactive protein (hs-CRP), was elevated in T3 (1.62 ± 7.35 mg/L) relative to T1 and T2 (*p* = 0.004). Thyroid-stimulating hormone (TSH) levels did not differ significantly across groups (*p* = 0.282).

### 3.2. Periprocedural and Discharge Characteristics

Table 3 presents procedural data and angiographic findings across KYN tertiles. Patients in the lowest KYN tertile (T1) were more frequently admitted due to ACS, while planned diagnostics for stable CAD predominated in T2 and T3 (*p* = 0.021). No significant atherosclerotic lesions were found in approximately 25% of patients across all groups, but multi-vessel disease (two- and three-vessel) was more prevalent in the higher tertiles (*p* = 0.022).

Treatment allocation differed significantly (*p* = 0.021), with PCI performed more often in T1 (55.4%), while patients in T2 were more likely to receive conservative management. CABG rates were comparable across tertiles. Most lesions were in the LAD/diagonal (38.3%) and RCA (34.0%), with no significant differences between groups. Post-procedural TIMI 3 flow was achieved in 94.5% of cases, although suboptimal flow (TIMI < 3) was slightly more frequent in T2 (*p* = 0.012). Periprocedural complications were rare and did not differ significantly between groups.

Table 4 displays the pharmacological therapies prescribed at hospital discharge stratified by KYN tertiles. Most patients received standard cardiovascular medications, including acetylsalicylic acid (ASA), ACE inhibitors, beta-blockers, and statins. Significant differences were observed in ASA and statin use, which were both less frequent in the highest KYN tertile (T3) compared to the lower groups (ASA: 85.4% in T3 vs. 93.9% in T1; *p* = 0.032; statins: 89.0% in T3 vs. 95.2% in T1; *p* = 0.043).

Patients in T3 were significantly more likely to be prescribed loop diuretics (30.5%) and mineralocorticoid receptor antagonists (18.9%) compared to those in T1 (9.7% and 6.1%, respectively; both *p* < 0.001 and *p* = 0.001), likely reflecting a higher burden of heart or renal dysfunction.

Use of vitamin K antagonists was also more common in T3 (12.8%) compared to T1 (4.2%, *p* = 0.007). The distribution of other medications, including clopidogrel, angiotensin receptor blockers, calcium channel blockers, fibrates, thiazides, and insulin, did not differ significantly between groups.

### 3.3. 10-Year Follow-Up Data

The median follow-up period was 10.2 years (min. 5.9 years; max. 10.3 years). Table 5 summarizes long-term clinical outcomes across KYN concentration tertiles. The incidence of all-cause mortality over the follow-up period differed significantly between groups, increasing progressively with higher KYN levels (17.6% in T1, 28.2% in T2, and 42.9% in T3; *p* < 0.001), indicating a strong association between elevated KYN and long-term mortality risk. For T2, HR was 1.69 with 95% CI 1.06–2.70, *p* = 0.026, and for T3, HR was 2.86, 95% CI 1.85–4.41, *p* < 0.001 (versus T1).

**Figure 2:**
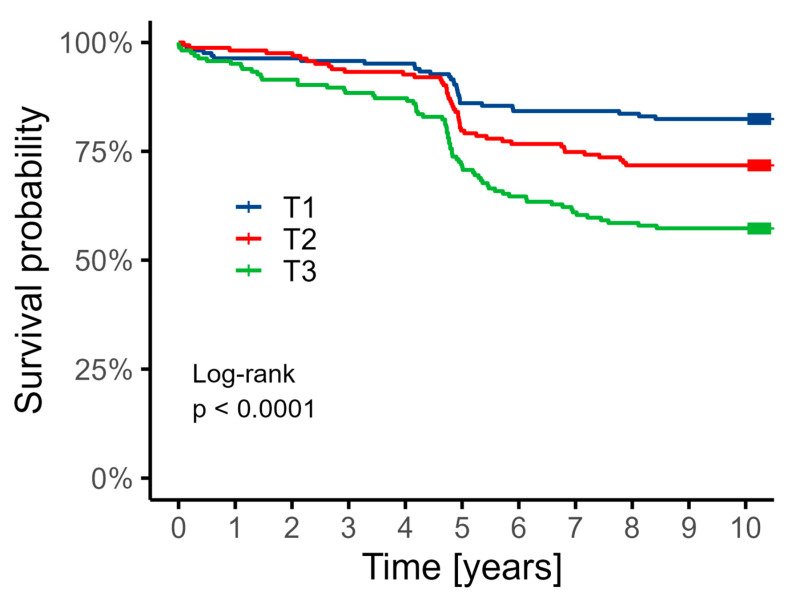
Kaplan–Meier curve showing survival depending on the tertile of KYN concentrations. The distribution of individual variables was compared between subgroups based on the tertiles.

In contrast, the rates of MI, stroke, CABG, and PCI were comparable across tertiles, with no statistically significant differences observed (all *p* > 0.05). While stroke appeared numerically less frequent in the highest tertile (2.5%), this trend did not reach statistical significance (*p* = 0.188) (Figure 2).

### 3.4. Cox Analysis

Although presented here within the final multivariable model, the analysis of KYN and related pathway metabolites was performed as the first step to explore whether any of these biomarkers might have prognostic significance. Univariable Cox analyses are provided in the Supplementary Table S1.

Table 6 presents the results of multivariable Cox regression analysis identifying factors associated with death over the 10-year follow-up period. Significant predictors of mortality included older age, with patients aged 65–75 years (HR = 3.54, *p* = 0.004) and 75–90 years (HR = 9.62, *p* < 0.001) demonstrating a substantially elevated risk compared to younger individuals. Other independent risk factors were diabetes (HR = 1.62, *p* = 0.025), previous MI (HR = 1.61, *p* = 0.017), and CKD (HR = 2.34, *p* < 0.001). The indication for coronary angiography due to STEMI was also associated with increased mortality (HR = 1.74, *p* = 0.043). In contrast, higher LVEF conferred a protective effect, with significantly lower hazard ratios observed in the 40–50% (HR = 0.41, *p* = 0.004), 50–60% (HR = 0.52, *p* = 0.031), and >60% (HR = 0.34, *p* = 0.003) subgroups. Importantly, KYN concentration emerged as an independent predictor of long-term mortality (HR = 1.79, *p* < 0.001).

## 4. Discussion

The present study investigates the association between KYN levels and long-term all-cause mortality in patients undergoing coronary angiography. Our findings reveal that elevated KYN concentrations are independently predictive of 10-year mortality, underscoring the potential role of the kynurenine pathway in CVD prognosis.

The kynurenine pathway is the primary route of tryptophan metabolism, leading to the production of several bioactive metabolites, including KYN, KYNA, and quinolinic acid (QA). Activation of this pathway has been implicated in various pathophysiological processes relevant to CVD, such as inflammation, oxidative stress, and endothelial dysfunction. Notably, KYN and its metabolites can modulate immune responses and vascular tone, potentially contributing to atherogenesis and cardiovascular events [19,20].

Our results align with prior research indicating a link between the kynurenine pathway and cardiovascular outcomes. For instance, the Hordaland Health Study demonstrated that elevated plasma levels of KYN and a higher kynurenine-to-tryptophan ratio were associated with increased risks of all-cause and CVD mortality [21]. This study followed over 7000 individuals for a median of 14 years. In this cohort, plasma KYN, anthranilic acid, and 3-HK levels were independently associated with increased all-cause mortality, while higher TRP and xanthurenic acid levels showed a protective association. Notably, the kynurenine/tryptophan ratio (KTR), an established marker of IDO activity and a surrogate of interferon-γ-mediated immune activation, was among the strongest predictors of mortality. After multivariate adjustment for conventional cardiovascular risk factors (age, sex, smoking, eGFR, BMI), individuals in the highest KTR quartile had a 60% higher risk of all-cause mortality (HR: 1.60; 95% CI: 1.32–1.94), compared to those in the lowest quartile. When analyzing cause-specific mortality, KTR, kynurenine, and 3-HK remained significantly associated with CVD mortality, suggesting a pivotal role of inflammatory activation and tryptophan catabolism in long-term cardiovascular risk. These data support the findings from the KORONEF registry and suggest that elevated KYN levels not only reflect an ongoing immune activation state but also directly contribute to disease progression and adverse outcomes, particularly in CVD.

Similarly, studies have reported that increased KYN concentrations correlate with adverse cardiac remodeling and poor prognosis in other groups. Further support for the cardiovascular relevance of the kynurenine pathway comes from a study in patients with primary hyperparathyroidism (pHPT), a population characterized by low-grade inflammation and elevated cardiovascular risk. In a cross-sectional analysis of 136 patients from the Eplerenone in Primary Hyperparathyroidism (EPATH) trial, investigators evaluated the association between inflammatory biomarkers—including CRP, KYN, quinolinic acid (QUIN), and TRP—and echocardiographic indicators of adverse cardiac remodeling. Multivariable regression analyses revealed that KYN and QUIN levels were significantly associated with key structural and functional markers of diastolic dysfunction. Specifically, higher KYN concentrations were independently related to increased left atrial volume index (LAVI; β = 0.256, *p* = 0.005) and E/e′ ratio (β = 0.221, *p* = 0.022), while QUIN was associated with all three parameters assessed: left ventricular mass index (LVMI; β = 0.270, *p* = 0.007), LAVI (β = 0.213, *p* = 0.044), and E/e′ (β = 0.292, *p* = 0.006). Interestingly, TRP itself showed no such associations. These findings indicate that activation of the KYN pathway may play a mechanistic role in structural cardiac remodeling and diastolic dysfunction, even in the absence of overt coronary artery disease. While our study focused on mortality as a primary endpoint, the observed links between KYN and myocardial remodeling support the plausibility of direct cardiotoxic or remodeling effects mediated by KYN derivatives, reinforcing their potential as prognostic markers and therapeutic targets in cardiovascular risk stratification [22].

In a study by Lund et al., elevated plasma KYN, 3-HK, and QA concentrations, as well as increased KTR levels and HK/xanthurenic acid (XA) ratios, were independently associated with higher all-cause mortality in patients with established heart failure, regardless of the presence of CAD [23]. Notably, HR for QA was 1.80 (*p* = 0.013), while for KTR, it was 1.55 (*p* = 0.009), underscoring the strength of these associations. These findings emphasize the broader involvement of the tryptophan–kynurenine pathway. not only in inflammation and immune modulation but also in structural and functional cardiac deterioration. Interestingly, XA appeared protective, being inversely related to mortality in all groups assessed, suggesting its role as a potential counter-regulator within this metabolic cascade.

Also, a recent large-scale prospective study from the CKD-REIN cohort further reinforces the adverse prognostic role of KYN in CVD, particularly in patients with CKD. El Chamieh et al. demonstrated that elevated serum-free KYN levels were independently associated with an increased risk of both fatal and nonfatal cardiovascular events, especially those classified as non-atheromatous, as well as cardiovascular mortality [24]. Notably, the association persisted even after adjusting for eGFR, serum-free TRP levels, other uremic toxins, and traditional cardiovascular risk factors. The adjusted HR for cardiovascular mortality with a doubling of serum-free KYN was 1.64 (95% CI: 1.10–2.40), and for non-atheromatous cardiovascular events, it was 1.26 (95% CI: 1.03–1.50). These findings are particularly relevant to our cohort, in which we observed a similarly strong and graded association between KYN levels and all-cause mortality over a 10-year follow-up. While the CKD-REIN cohort focused on patients with moderate to advanced CKD, the pathophysiological implications of KYN—such as its capacity to induce endothelial dysfunction, oxidative stress, and inflammation—may extend to broader cardiovascular populations, including those with CAD and mixed comorbidities such as in the KORONEF registry.

Moreover, our findings align with the EPIC-Norfolk prospective study, which robustly demonstrated that increased plasma KYN concentrations and elevated KTR are significantly associated with both incident and fatal cardiovascular events in a large, community-based cohort followed over two decades. In multivariable-adjusted Cox models, higher levels of KYN were associated with an increased risk of cardiovascular disease (HR 1.33; 95% CI: 1.19–1.49), while elevated KTR also conferred higher risk (HR 1.24; 95% CI: 1.14–1.35), independent of traditional risk factors and CRP levels [25]. Notably, KYN and its metabolic ratio with TRP also predicted fatal cardiovascular events, including fatal MI and peripheral artery disease. These results support the pro-inflammatory and immunomodulatory roles of KYN pathway activation, likely reflecting underlying IDO activity driven by low-grade inflammation. Importantly, these associations remained significant even after adjusting for CRP, implying that kynurenine-related mechanisms may go beyond classical inflammation markers.

Our study extends these findings by providing long-term follow-up data (10-year follow-up), reinforcing the prognostic significance of KYN in a diverse patient population undergoing coronary angiography.

The mechanisms by which elevated KYN levels contribute to increased mortality in cardiovascular populations are complex and multifactorial, involving both immune and vascular pathways. One of the central mediators of KYN’s biological activity is the aryl hydrocarbon receptor (AhR), a ligand-activated transcription factor expressed in various cell types, including vascular endothelial cells, immune cells, and cardiomyocytes. Upon binding of KYN, AhR translocates to the nucleus and induces the expression of genes involved in inflammation, oxidative stress, and thrombosis, thereby promoting vascular dysfunction and a pro-atherogenic environment. Persistent activation of AhR has also been linked to impaired endothelial nitric oxide synthase (eNOS) activity, reduced nitric oxide bioavailability, and enhanced leukocyte adhesion—all of which facilitate the progression of endothelial injury and plaque instability [26]. Moreover, downstream metabolites of KYN, such as 3-HK and QA, further amplify cardiovascular risk through pro-oxidant and cytotoxic properties. 3-HK can generate reactive oxygen species (ROS) and induce lipid peroxidation, damaging vascular cells and promoting apoptosis [12]. QA, a neurotoxic metabolite also implicated in peripheral vascular pathology, has been shown to activate N-methyl-D-aspartate (NMDA) receptors on endothelial cells, disrupting intracellular calcium homeostasis and exacerbating oxidative stress. These processes contribute directly to endothelial dysfunction, a critical early step in the development of atherosclerosis, as well as vascular remodeling and stiffness, which have been independently linked to adverse cardiovascular outcomes. Furthermore, activation of the KYN pathway is tightly regulated by inflammatory cytokines, particularly interferon-γ and tumor necrosis factor-α, which induce IDO, the rate-limiting enzyme of the pathway. In this context, elevated KYN levels may reflect a state of chronic low-grade inflammation that predisposes individuals to atherosclerotic plaque progression, microvascular dysfunction, and impaired myocardial repair mechanisms following ischemic injury [12,19]. Taken together, these mechanisms provide a biological rationale for the observed association between higher KYN levels and increased long-term mortality, as reported in our study and corroborated by several recent prospective cohorts.

Emerging research is also beginning to explore how modifiable lifestyle factors, such as physical activity, may influence KYN pathway activation and its downstream cardiovascular effects. A recent interventional study in patients with CAD (ClinicalTrials.gov: NCT06579807) aims to evaluate both the acute and chronic impact of aerobic exercise on KYN pathway metabolites, inflammatory cytokines, and vascular function. The study will assess carotid intima–media thickness, endothelial function, and biomarkers such as TRP, KYN, KYNA, and QA in response to structured exercise over 12 weeks. By examining the link between exercise-induced modulation of the KYN pathway and vascular inflammation, this trial may offer mechanistic insights into non-pharmacological strategies to mitigate cardiovascular risk. Its findings will complement our current results by addressing whether KYN pathway activation is reversible or modifiable through behavioral interventions in CAD patients [27].

The independent association between KYN levels and long-term mortality suggests that KYN could serve as a valuable biomarker for risk stratification in patients undergoing coronary angiography. Measuring KYN concentrations may aid in identifying individuals at higher risk for adverse outcomes, potentially guiding more personalized therapeutic interventions. Unfortunately, although our findings support an association between elevated plasma kynurenine levels and 10-year all-cause mortality, they do not establish a causal relationship. The kynurenine pathway is strongly influenced by pro-inflammatory cytokines such as interferon-γ and TNF-α, and elevated KYN concentrations likely reflect ongoing immune activation and metabolic stress rather than direct cardiovascular toxicity. This interpretation is consistent with the growing body of literature suggesting that kynurenine acts primarily as a prognostic biomarker rather than a confirmed therapeutic target. As such, our results should be viewed in the context of systemic pathophysiological processes rather than isolated vascular mechanisms. Future mechanistic and interventional studies are needed to determine whether targeting the kynurenine pathway could modify cardiovascular outcomes.

Nevertheless, beyond its prognostic value, the kynurenine pathway has attracted some interest as a potential therapeutic target, particularly through modulation of its enzymatic regulators. Several pharmacological strategies are currently under investigation, including indoleamine 2,3-dioxygenase 1 (IDO1) inhibitors, kynurenine 3-monooxygenase (KMO) inhibitors, and tryptophan mimetics, primarily in the context of cancer, neurodegeneration, and chronic inflammatory diseases [28,29,30]. Although these agents have not yet been studied in cardiovascular populations, they may provide tools to test the causal relevance of kynurenine pathway activation in human vascular pathology. Future interventional studies will be essential to determine whether modifying this pathway could translate into clinical benefit for patients with cardiometabolic disease.

## 5. Study Limitations

This study has several limitations. First, the study cohort included not only patients with CAD but also individuals referred for coronary angiography for other indications, resulting in a clinically heterogeneous population. Second, only clopidogrel was used as antiplatelet therapy, without newer P2Y12 inhibitors, which may have influenced outcomes. Third, kynurenine and related metabolite levels were assessed only once at baseline, without follow-up measurements to assess longitudinal changes or intra-individual variability.

## 6. Conclusions

In this 10-year follow-up study from the KORONEF registry, elevated plasma KYN concentration emerged as a significant and independent predictor of all-cause mortality in patients undergoing coronary angiography. These findings support the potential role of KYN as a biomarker of long-term cardiovascular risk, reflecting systemic pathophysiology beyond conventional imaging and clinical factors.

## Figures and Tables

**Table 1:** Baseline characteristics based on KYN concentration.

Parameter	Total N = 492	Tertile 1 N = 165	Tertile 2 N = 163	Tertile 3 N = 164	*p*
Females	184 (37.4%)	66 (40.0%)	50 (30.7%)	68 (41.5%)	0.092
Age [years]	64.4 (9.8)	62.17 (9.45)	64.98 (9.34)	66.04 (10.37)	<0.001
BMI [kg/m^2^]	27.9 (4.3)	27.45 (4.57)	28.00 (3.84)	28.28 (4.34)	0.126
Arterial hypertension	366 (74.8%)	115 (69.7%)	123 (76.4%)	128 (78.5%)	0.157
Dyslipidemia	225 (46.0%)	78 (47.3%)	68 (42.2%)	79 (48.5%)	0.490
Diabetes	126 (25.8%)	42 (25.5%)	35 (21.7%)	49 (30.1%)	0.229
Obesity	147 (30.1%)	44 (26.7%)	53 (32.9%)	50 (30.7%)	0.459
Previous MI	154 (31.5%)	37 (22.4%)	54 (33.5%)	63 (38.7%)	0.005
Previous stroke	32 (6.6%)	5 (3.0%)	13 (8.1%)	14 (8.6%)	0.078
Peripheral artery disease	24 (4.9%)	4 (2.4%)	11 (6.8%)	9 (5.5%)	0.166
Chronic kidney disease	47 (9.6%)	6 (3.6%)	11 (6.8%)	30 (18.4%)	<0.001
Previous CABG	21 (4.3%)	2 (1.2%)	9 (5.6%)	10 (6.1%)	0.055
Previous PCI	103 (21.1%)	27 (16.4%)	32 (19.9%)	44 (27.0%)	0.056
Echocardiography results					
LV ejection fraction [%]	52.5 (11.5)	54.33 (10.3)	52.30 (11.56)	50.97 (12.49)	0.096
RVSP [mmHg]	38.9 (10.3)	39.14 (7.36)	36.22 (11.37)	40.38 (10.96)	0.558
TAPSE [mm]	17.4 (4.2)	21.00 (0.21)	22.50 (0.71)	14.50 (2.43)	0.029

Results presented as mean (standard deviation): BMI—body mass index; MI—myocardial infarction; CABG—coronary artery bypass grafting; PCI—percutaneous coronary intervention; LV—left ventricular; RVSP—right ventricular systolic pressure; TAPSE—tricuspid annular plane systolic excursion.

**Table 2:** Biochemical tests based on KYN concentration.

Parameter	Total N = 492	Tertile 1 N = 165	Tertile 2 N = 163	Tertile 3 N = 164	*p*
Hemoglobin [g/dL]	13.87 (1.39)	13.87 (1.40)	13.92 (1.32)	13.82 (1.45)	0.854
Glucose [mg/dL]	116.42 (39.56)	116.69 (34.69)	111.38 (34.65)	121.05 (47.58)	0.105
Creatinine [mg/dL]	0.99 (0.57)	0.85 (0.20)	0.93 (0.19)	1.19 (0.91)	<0.001
eGFR [mL/min]	81.68 (24.22)	90.37 (23.26)	83.77 (21.81)	70.76 (23.41)	<0.001
K^+^ [mmol/L]	4.37 (0.46)	4.28 (0.44)	4.36 (0.40)	4.48 (0.51)	0.001
Na^+^ [mmol/L]	140.72 (2.84)	140.88 (2.84)	141.07 (2.63)	140.20 (2.99)	0.027
Total cholesterol [mg/dL]	183.23 (51.13)	197.28 (59.51)	177.36 (45.80)	174.62 (43.51)	<0.001
LDL [mg/dL]	109.60 (44.49)	118.87 (51.67)	104.93 (41.39)	104.79 (37.81)	0.020
HDL [mg/dL]	53.47 (17.45)	55.70 (18.84)	53.73 (16.94)	50.89 (16.17)	0.036
Triglycerides [mg/dL]	315.01 (39.53)	138.91 (86.85)	667.98 (77.78)	139.23 (77.43)	0.234
TSH [μIU/mL]	2.14 (4.84)	2.68 (8.23)	1.86 (2.01)	1.91 (1.51)	0.282
hs-CRP [mg/L]	1.16 (4.94)	0.93 (3.58)	0.92 (2.50)	1.62 (7.35)	0.004

Results presented as mean (standard deviation); HDL—high-density lipoprotein; LDL—low-density lipoprotein; TSH—thyroid-stimulating hormone; hsCRP—high sensitivity C-reactive protein.

**Table 3:** Periprocedural data based on KYN concentration.

Parameter	Total N = 492	Tertile 1 N = 165	Tertile 2 N = 163	Tertile 3 N = 164	*p*
Coronary angiography indications					
Planned coronary artery disease diagnostics	290 (59.4%)	78 (47.3%)	106 (66.2%)	106 (65.0%)	0.021
Acute coronary syndrome	170 (34.6%)	78 (47.3%)	45 (27.6%)	47 (28.7%)	
Heart failure diagnostics	4 (0.8%)	2 (1.2%)	0 (0.0%)	2 (1.2%)	
Pacemaker/ICD qualification	4 (0.8%)	2 (1.2%)	0 (0.0%)	2 (1.2%)	
Cardiovascular surgery (heart valve defect, ascending aorta aneurysm)	24 (4.9%)	7 (4.2%)	9 (5.6%)	8 (4.9%)	
Coronary angiography results					
No significant atherosclerotic lesions *	122 (25.0%)	38 (23.0%)	41 (25.6%)	43 (26.4%)	0.022
One-vessel disease	140 (28.7%)	60 (36.4%)	45 (28.1%)	35 (21.5%)	
Two-vessel disease	137 (28.1%)	40 (24.2%)	45 (28.1%)	52 (31.9%)	
Three-vessel disease	67 (13.7%)	20 (12.1%)	23 (14.4%)	24 (14.7%)	
Left main stem	22 (4.5%)	7 (4.2%)	6 (3.8%)	9 (5.5%)	
Qualification for revascularization					
Pharmacological treatment	156 (34.3%)	43 (27.4%)	58 (39.7%)	55 (36.2%)	0.021
PCI	208 (45.7%)	87 (55.4%)	53 (36.3%)	68 (44.7%)	
CABG	91 (20.0%)	27 (17.2%)	35 (24.0%)	29 (19.1%)	
Location of lesions treated by PCI					
Left main stem	1 (0.4%)	0 (0.0%)	1 (1.5%)	0 (0.0%)	0.034
Left anterior descending artery/ diagonal branches	90 (38.3%)	38 (41.3%)	24 (36.4%)	28 (36.4%)	
Left circumflex artery/marginal branches	58 (24.7%)				
Intermediate artery	4 (1.7%)	2 (2.2%)	1 (1.5%)	1 (1.3%)	
Right coronary artery	80 (34.0%)	33 (35.9%)	22 (33.3%)	25 (32.5%)	
Venous graft	2 (0.9%)	0 (0.0%)	1 (1.5%)	1 (1.3%)	
TIMI after PCI					
0	10 (4.2%)	0 (0.0%)	6 (9.0%)	4 (5.3%)	0.012
1	2 (0.8%)	1 (1.1%)	1 (1.5%)	0 (0.0%)	
2	1 (0.4%)	1 (1.1%)	0 (0.0%)	0 (0.0%)	
3	223 (94.5%)	91 (97.8%)	60 (89.6%)	72 (94.7%)	
Periprocedural complications (PCI)					
No reflow/slow reflow	8 (3.8%)	0 (0.0%)	4 (2.8%)	4 (2.5%)	0.116
Stent thrombosis	1 (0.5%)	1 (0.6%)	0 (0.0%)	0 (0.0%)	0.998

* no atherosclerotic lesions or lesions < 40%; TIMI—thrombolysis in myocardial infarction; PCI—percutaneous coronary intervention; CABG—coronary artery bypass grafting; MI—myocardial infarction; ICD—implantable cardioverter defibrillator.

**Table 4:** Medications at discharge based on KYN concentration.

Parameter	Total N = 492	Tertile 1 N = 165	Tertile 2 N = 163	Tertile 3 N = 164	*p*
Acetylsalicylic acid	442 (90.0%)	155 (93.9%)	147 (90.7%)	140 (85.4%)	0.032
Clopidogrel	301 (61.3%)	111 (67.3%)	91 (56.2%)	99 (60.4%)	0.114
ACE inhibitor	426 (86.8%)	143 (86.7%)	144 (88.9%)	139 (84.8%)	0.545
Angiotensin antagonist	21 (4.3%)	10 (6.1%)	6 (3.7%)	5 (3.0%)	0.365
Beta-blocker	452 (92.1%)	152 (92.1%)	154 (95.1%)	146 (89.0%)	0.131
Ca-blocker	124 (25.3%)	39 (23.6%)	43 (26.5%)	42 (25.6%)	0.826
Statin	457 (93.1%)	157 (95.2%)	154 (95.1%)	146 (89.0%)	0.043
Fibrate	17 (3.5%)	5 (3.0%)	3 (1.9%)	9 (5.5%)	0.186
Loop diuretic	89 (18.1%)	16 (9.7%)	23 (14.2%)	50 (30.5%)	<0.001
Thiazide	50 (10.2%)	13 (7.9%)	19 (11.7%)	18 (11.0%)	0.474
Mineralocorticoid receptor antagonist	59 (12.0%)	10 (6.1%)	18 (11.1%)	31 (18.9%)	0.001
Vitamin K antagonist	37 (7.5%)	7 (4.2%)	9 (5.6%)	21 (12.8%)	0.007
Insulin	44 (9.0%)	17 (10.3%)	10 (6.2%)	17 (10.4%)	0.316

ACE—angiotensin-converting enzyme.

**Table 5:** The outcomes in 10-year follow-up.

Endpoint	Total N = 492	Tertile 1 N = 165	Tertile 2 N = 163	Tertile 3 N = 164	*p*
Death	145 (29.5%)	29 (17.6%)	46 (28.2%)	70 (42.9%)	<0.001
MI	56 (11.4%)	17 (10.3%)	21 (12.9%)	18 (11.0%)	0.751
Stroke	24 (4.9%)	11 (6.7%)	9 (5.5%)	4 (2.5%)	0.188
CABG	37 (7.5%)	12 (7.3%)	14 (8.6%)	11 (6.7%)	0.810
PCI	81 (16.5%)	24 (14.5%)	29 (17.8%)	28 (17.2%)	0.701

MI—myocardial infarction; CABG—coronary artery bypass grafting; PCI—percutaneous coronary intervention.

**Table 6:** Factors predicting death in the 10-year follow-up—multivariable Cox analysis.

	Study Population N = 492 (%)		
Parameter	HR	95% CI	*p*-Value
Age:			
65–75 years	3.54	1.51, 8.12	0.004
75–90 years	9.62	4.21, 21.8	<0.001
Diabetes	1.62	1.01, 2.44	0.025
Previous myocardial infarction	1.61	1.03, 2.45	0.017
Chronic kidney disease	2.34	1.44, 3.81	<0.001
Coronary angiography indications			
STEMI	1.74	1.05, 3.03	0.043
Left ventricular ejection fraction			
[40, 50]	0.41	0.29, 0.74	0.004
[50, 60]	0.52	0.30, 0.95	0.031
>60	0.34	0.24, 0.72	0.003
KYN	1.79	1.15, 2.44	<0.001

HR = hazard ratio; CI = confidence interval; STEMI—ST-elevation myocardial infarction; KYN—kynurenine.

## Data Availability

Data are available from the corresponding author on request.

## Supplementary Materials

The following supporting information can be downloaded at https://www.mdpi.com/article/10.3390/biomedicines13051123/s1: Supplementary Table S1. Table S1: Univariable Cox regression for death in the whole population.

## Abbreviations

The following abbreviations are used in this manuscript:

3-HAA	3-Hydroxyanthranilic acid
3-HK	3-Hydroxykynurenine
AA	Anthranilic acid
ACS	Acute coronary syndrome
ASA	Acetylsalicylic acid
AhR	Aryl hydrocarbon receptor
BMI	Body mass index
CABG	Coronary artery bypass grafting
CAD	Coronary artery disease
CCS	Chronic coronary syndrome
CK	Creatine kinase
CK-MB	Creatine kinase-MB isoenzyme
CKD	Chronic kidney disease
COPD	Chronic obstructive pulmonary disease
CRP	C-reactive protein
DAPT	Dual antiplatelet therapy
E/e′	Ratio of early mitral inflow velocity to mitral annular early diastolic velocity
ECG	Electrocardiogram
HDL	High-density lipoprotein
HPLC	High-performance liquid chromatography
Hgb	Hemoglobin
ICD	Implantable cardioverter defibrillator
IDO	Indoleamine 2,3-dioxygenase
K+	Potassium ion
KP	Kynurenine pathway
KTR	Kynurenine/tryptophan ratio
KYN	Kynurenine
KYNA	Kynurenic acid
LAD	Left anterior descending artery
LAVI	Left atrial volume index
LDL	Low-density lipoprotein
LVEF	Left ventricular ejection fraction
LVMI	Left ventricular mass index
MI	Myocardial infarction
NT-proBNP	N-terminal prohormone of brain natriuretic peptide
PCI	Percutaneous coronary intervention
PLT	Platelet
QA	Quinolinic acid
RBC	Red blood cell
ROS	Reactive oxygen species
RVSP	Right ventricular systolic pressure
STEMI	ST-elevation myocardial infarction
TAPSE	Tricuspid annular plane systolic excursion
TG	Triglyceride
TIMI	Thrombolysis in myocardial infarction (flow grade)
TRP	Tryptophan
TSH	Thyroid-stimulating hormone
UA	Unstable angina
WBC	White blood cell
eGFR	Estimated glomerular filtration rate
hs-CRP	High-sensitivity C-reactive protein
pHPT	Primary hyperparathyroidism
