# Supplementary material for: Kynurenine as a Predictor of Long-Term Mortality: A 10-Year Follow-Up from the KORONEF Registry
Source: Biomedicines. 2025 May 6;13(5):1123. doi: 10.3390/biomedicines13051123 (PMC12109461; doi:10.3390/biomedicines13051123)
Supplement: Supplementary file 1 [file biomedicines-13-01123-s001.zip › biomedicines-3605223-supplementary.pdf]

Supplementary Table S1. Univariable Cox regression

| Characteristic | HR[1] | 95% CI[1] | p-value |
|---|---|---|---|
| **Sex** | | | |
| Female | — | — | |
| Male | 1.02 | 0.73, 1.43 | 0.9 |
| **Age - groups** | | | |
| (30,55] | — | — | |
| (55,60] | 1.81 | 0.83, 3.93 | 0.13 |
| (60,65] | 1.45 | 0.63, 3.31 | 0.4 |
| (65,75] | 3.67 | 1.80, 7.48 | <0.001 |
| (75,90] | 8.71 | 4.29, 17.7 | <0.001 |
| **BMI** | | | |
| <25 | — | — | |
| [25,29.9] | 0.94 | 0.60, 1.48 | 0.8 |
| [30,34.9] | 0.76 | 0.43, 1.34 | 0.3 |
| ≥35 | 0.66 | 0.26, 1.71 | 0.4 |
| **Diabetes** | | | |
| no | — | — | |
| yes | 2.01 | 1.43, 2.82 | <0.001 |
| **Arterial hypertension** | | | |
| no | — | — | |
| yes | 1.15 | 0.78, 1.70 | 0.5 |
| **Smoking** | | | |
| no | — | — | |
| yes | 0.67 | 0.45, 1.00 | 0.048 |
| **Obesity** | | | |
| no | — | — | |
| yes | 1.03 | 0.72, 1.46 | 0.9 |
| **Dyslipidemia** | | | |
| no | — | — | |
| yes | 0.65 | 0.46, 0.91 | 0.011 |
| **Prior MI** | | | |
| no | — | — | |

| Characteristic | HR[1] | 95% CI[1] | p-value |
|---|---|---|---|
| yes | 2.02 | 1.45, 2.80 | <0.001 |
| **Prior stroke** | | | |
| no | — | — | |
| yes | 2.79 | 1.72, 4.52 | <0.001 |
| **Peripheral artery disease** | | | |
| no | — | — | |
| yes | 1.72 | 0.93, 3.19 | 0.082 |
| **Hemodialysis** | | | |
| no | — | — | |
| yes | 0.00 | 0.00, Inf | >0.9 |
| **Chronic kidney disease** | | | |
| no | — | — | |
| yes | 2.71 | 1.78, 4.12 | <0.001 |
| **Prior CABG** | | | |
| no | — | — | |
| yes | 1.81 | 0.95, 3.45 | 0.070 |
| **Prior PCI** | | | |
| no | — | — | |
| yes | 1.30 | 0.89, 1.90 | 0.2 |
| **STEMI** | | | |
| no | — | — | |
| yes | 1.10 | 0.71, 1.70 | 0.7 |
| **NSTEMI** | | | |
| no | — | — | |
| yes | 1.27 | 0.81, 2.01 | 0.3 |
| **UA** | | | |
| no | — | — | |
| yes | 1.15 | 0.67, 1.97 | 0.6 |
| **Cardiac arrest** | | | |
| no | — | — | |
| yes | 3.36 | 1.38, 8.21 | 0.008 |
| **AF** | | | |

| Characteristic | HR[1] | 95% CI[1] | p-value |
|---|---|---|---|
| no | — | — | |
| yes | 2.66 | 1.68, 4.24 | <0.001 |
| **Coronary artery disease advancement** | | | |
| 3-VD | — | — | |
| LM | 1.25 | 0.57, 2.71 | 0.6 |
| **BMS No** | | | |
| 0 | — | — | |
| 1 | 1.00 | 0.68, 1.46 | >0.9 |
| 2 | 1.65 | 0.83, 3.26 | 0.2 |
| 3 | 1.25 | 0.17, 8.96 | 0.8 |
| **DES No** | | | |
| 0 | — | — | |
| 1 | 0.63 | 0.38, 1.06 | 0.083 |
| 2 | 0.74 | 0.18, 2.99 | 0.7 |
| 3 | 0.00 | 0.00, Inf | >0.9 |
| **POBA** | | | |
| 0 | — | — | |
| 1 | 0.86 | 0.55, 1.35 | 0.5 |
| 2 | 2.78 | 0.39, 19.9 | 0.3 |
| **TIMI after PCI** | | | |
| 0 | — | — | |
| 1 | 0.74 | 0.09, 6.34 | 0.8 |
| 2 | 0.00 | 0.00, Inf | >0.9 |
| 3 | 0.43 | 0.17, 1.08 | 0.072 |
| **Indications for coronary angiography** | | | |
| CAD | — | — | |
| NSTEMI | 1.10 | 0.68, 1.77 | 0.7 |
| STEMI | 1.21 | 0.77, 1.92 | 0.04 |
| UA | 0.33 | 0.12, 0.89 | 0.028 |
| **%DS renal artery** | | | |
| <50% | — | — | |

| Characteristic | HR[1] | 95% CI[1] | p-value |
|---|---|---|---|
| ≥50% | 1.41 | 0.81, 2.46 | 0.2 |
| **Echo EF** | | | |
| ≤40 | — | — | |
| (40,50] | 0.49 | 0.31, 0.78 | 0.002 |
| (50,60] | 0.44 | 0.27, 0.73 | 0.002 |
| >60 | 0.27 | 0.16, 0.46 | <0.001 |
| **hsCRP** | | | |
| ≤0.1 | — | — | |
| (0.1,0.2] | 1.08 | 0.63, 1.85 | 0.8 |
| (0.2,0.5] | 1.12 | 0.68, 1.83 | 0.7 |
| (0.5,82] | 1.55 | 0.95, 2.53 | 0.080 |
| **LDL chol** | | | |
| ≤100 | — | — | |
| (100,129] | 0.55 | 0.34, 0.88 | 0.014 |
| (129,159] | 0.72 | 0.45, 1.15 | 0.2 |
| (159,465] | 0.83 | 0.49, 1.40 | 0.5 |
| **Glucose** | | | |
| ≤80 | — | — | |
| (80,100] | 0.95 | 0.34, 2.64 | >0.9 |
| (100,140] | 1.27 | 0.46, 3.50 | 0.6 |
| (140,200] | 1.32 | 0.45, 3.89 | 0.6 |
| >200 | 2.74 | 0.89, 8.41 | 0.078 |
| **eGFR** | | | |
| ≤60 | — | — | |
| >60 | 0.29 | 0.21, 0.42 | <0.001 |
| **TRP (uM)** | 1.00 | 0.99, 1.02 | 0.5 |
| **KYN (uM)** | 1.29 | 1.15, 1.44 | <0.001 |
| **3-HKYN (nM)** | 1.01 | 1.01, 1.02 | <0.01 |
| **KYNA (nM)** | 1.00 | 0.99, 1.01 | >0.9 |
| **3-HAA (nM)** | 1.00 | 0.99, 1.01 | >0.9 |
| **AA (nM)** | 1.00 | 0.99, 1.01 | 0.7 |
| **KYN (uM) / TRP (uM)** | 16.4 | 1.16, 233 | 0.038 |
